# Diagnostic Potential of Alternations of Bile Acid Profiles in the Plasma of Patients with Huntington’s Disease

**DOI:** 10.3390/metabo14070394

**Published:** 2024-07-20

**Authors:** Ping-I Chiang, Kuo-Hsuan Chang, Hsiang-Yu Tang, Yih-Ru Wu, Mei-Ling Cheng, Chiung-Mei Chen

**Affiliations:** 1 Department of Medical Education, Taipei Tzu Chi Hospital, Buddhist Tzu Chi Medical Foundation, New Taipei 231, Taiwan; pc20525386@gmail.com; 2 Department of Neurology, Chang Gung Memorial Hospital, Linkou Medical Center, College of Medicine, Chang Gung University, Taoyuan 333, Taiwan; 3 Metabolomics Core Laboratory, Healthy Aging Research Center, Chang Gung University, Taoyuan 333, Taiwan; 4 Clinical Metabolomics Core Laboratory, Chang Gung Memorial Hospital, Taoyuan-333, Taiwan; 5 Department of Biomedical Sciences, Chang Gung University, Taoyuan 333, Taiwan

**Keywords:** Huntington’s disease, bile acid, glycochenodeoxycholic acid, glycoursodeoxycholic acid, isolithocholic acid

## Abstract

Huntington’s disease (HD) is characterized by progressive involuntary chorea movements and cognitive decline. Recent research indicates that metabolic disturbance may play a role in its pathogenesis. Bile acids, produced during cholesterol metabolism in the liver, have been linked to neurodegenerative conditions. This study investigated variations in plasma bile acid profiles among individuals with HD. Plasma levels of 16 primary and secondary bile acids and their conjugates were analyzed in 20 healthy controls and 33 HD patients, including 24 with symptoms (symHD) and 9 carriers in the presymptomatic stage (preHD). HD patients exhibited significantly higher levels of glycochenodeoxycholic acid (GCDCA) and glycoursodeoxycholic acid (GUDCA) compared to healthy controls. Conversely, isolithocholic acid levels were notably lower in the HD group. Neurotoxic bile acids (glycocholic acid (GCA) + glycodeoxycholic acid (GDCA) + GCDCA) were elevated in symHD patients, while levels of neuroprotective bile acids (ursodeoxycholic acid (UDCA) + GUDCA + tauroursodeoxycholic acid (TUDCA)) were higher in preHD carriers, indicating a compensatory response to early neuronal damage. These results underscore the importance of changes in plasma bile acid profiles in HD and their potential involvement in disease mechanisms. The identified bile acids (GCDCA, GUDCA, and isolithocholic acid) could potentially serve as markers to distinguish between HD stages and healthy individuals. Nonetheless, further research is warranted to fully understand the clinical implications of these findings and their potential as diagnostic or therapeutic tools for HD.

## 1. Introduction

Huntington’s disease (HD) is an inherited neurodegenerative disorder characterized by involuntary choreiform movements, cognitive deterioration, and psychiatric symptoms. The disease is caused by an unstable cytosine–adenine–guanine (CAG) trinucleotide expansion in exon 1 of huntingtin (*HTT*) gene [1]. The diagnosis of HD is confirmed by a targeted mutation analysis finding of a CAG trinucleotide expansion of ≥36 repeats in the *HTT* gene. Genetic testing is sensitive (98.8 percent) and specific (100 percent) for HD [2]. When the CAG expands beyond the normal range, it becomes unstable and the mutant HTT protein becomes prone to misfolding. This aberrant protein misfolds and forms harmful aggregates within cell nuclei and cytoplasm, disrupting normal cellular functions. These disruptions result in transcriptional dysregulation [3], oxidative stress, mitochondrial dysfunction, metabolic disturbances [4], and impaired proteasome activity [5], ultimately causing neuronal dysfunction and death [6]. These complex mechanisms alter the overall cellular metabolism, leading to changes in metabolite profiles. Researchers have investigated various metabolites in HD patients, including prothrombin, apolipoprotein, haptoglobin, amino acids, fatty acids, taurine, serotonin, and phosphatidylcholines [7,8,9,10]. A cross-sectional analysis of plasma and cerebrospinal fluid (CSF) metabolic markers in HD suggests that these alterations are not limited to the central nervous system (CNS) but also extend to peripheral tissues [11]. Therefore, identifying molecular markers in plasma may help track disease progression and therapeutic response in HD patients. Currently, there are no disease-modifying treatments for HD, and therefore, available therapeutic strategies remain primarily symptomatic. Most regimens target motor, cognitive, and psychiatric symptom management, including dopamine modulators such as Tetrabenazine and neuroleptics [12].

Bile acids (BAs) are cholesterol-derived molecules primarily synthesized in the liver, playing a crucial role in the excretion, absorption, and transport of lipids and sterols within the intestine and liver [13,14]. They regulate bile flow, facilitate the absorption of dietary fats and vitamins, and influence key enzymes involved in cholesterol homeostasis [13,14]. The two major pathways of BA synthesis are the classical and the alternative pathways [15]. While the classical pathway predominates in the liver, the BAs derived from 24 (S)-hydroxycholesterol in the brain mainly involve the alternative pathway [16]. Primary BAs, mainly cholic acids (CA) and chenodeoxycholic acid (CDCA), and their conjugate derivatives are formed primarily in the liver and further converted to secondary BAs, such as deoxycholic acid (DCA), lithocholic acid (LCA), glycodeoxycholic acid (GDCA), via deconjugation and dehydroxylation by specific gut microbiome enzymes [17,18]. Generally, most primary BAs (for example, CA and CDCA) are cytoprotective, while most gut bacteria-associated secondary BAs (for example, DCA, LCA, and GDCA) are considered cytotoxic.

Emerging evidence suggests that BAs serve as versatile signaling molecules, not only in peripheral tissues but also within the CNS [19]. Previous studies have proposed that alterations in BA metabolism are evident in various neurodegenerative conditions, such as Alzheimer’s disease (AD), Parkinson’s disease (PD), amyotrophic lateral sclerosis (ALS), and cerebrotendinous xanthomatosis (CTX) [20,21]. For example, an AD study of 1464 subjects found significantly lower serum concentrations of primary BAs and higher levels of secondary BAs in AD patients compared to cognitively normal older adults [22]. Ratios of DCA/CA and GDCA/CA, which reflect the conversion of primary to secondary BAs by gut bacteria, were strongly linked to cognitive decline [22]. In a PD study, lower serum levels of neuroprotective BAs (CDCA, CA, and UDCA) were significantly associated with mild cognitive impairment in PD [23]. These disorders are consistently associated with gut dysfunction [24,25,26,27]. Similarly, HD patients also display significant weight loss, with other gut-related symptoms, including diarrhea, leaky gut, dysphagia, and microbial dysbiosis [26]. Despite these findings, BA profiles in HD have not yet been explored, with existing research primarily focusing on the association with 24S-hydroxycholesterol (24OHC) and lipoprotein metabolism in HD [28,29].

In this study, we aim to elucidate if the profiles of BAs are altered in HD and their utility as prospective biomarkers for distinguishing presymptomatic (preHD) and symptomatic HD (symHD) individuals from healthy controls (HC).

## 2. Materials and Methods

### 2.1. Ethics Statement and Study Participants

This study was approved by the Institutional Review Board of Chang Gung Memorial Hospital, Linkou, Taiwan (ethical license No: 201601128A3C501, 1 November 2016 and 202100892A3, 21 October 2021). We obtained written informed consent from all participants.

### 2.2. Participants Recruitment and Plasma Preparation

Patients with symptomatic HD (symHD), presymptomatic HD (preHD), and gender- and age-matched healthy controls (HC) were recruited from the outpatient clinics, Department of Neurology, Chang Gung Memorial Hospital. HD diagnosis was confirmed through clinical features and genetic testing that revealed extended CAG repeats in exon 1 of the *HTT gene* [1]. Disease status was assessed using the Unified Huntington’s Disease Rating Scale (UHDRS) [30], which includes total motor scores ranging from 0 to 124, an independence scale from 100 to 10, and a functional capacity scale from 13 to 0, indicating normal to most severe disease status. A disease burden score was calculated using the formula (age × [CAG − 35.5]) [31]. Individuals carrying the *HTT* gene mutation without clinical symptoms and with a total motor score of zero were classified as preHD. All participants were free from significant cardiac or liver dysfunction, chronic renal function impairment autoimmune diseases, malignancies, systemic infection, stroke, or other neurodegenerative diseases, except HD. Additionally, they were advised to abstain from smoking, alcohol, and nutritional supplements for two weeks. Blood samples were collected into EDTA-containing tubes from participants who had fasted overnight for at least 8 h. The samples were centrifuged at 1500–2000× *g* for 20 min within one hour. The plasma (supernatant) was then transferred to a fresh 1.5 mL Eppendorf tube and stored at −80 °C before assessments.

### 2.3. Bile Acid Analysis with Liquid Chromatography–Mass Spectrometry (LCMS)

Lithocholic acid (LCA), deoxycholic acid (DCA), chenodeoxycholic acid (CDCA), ursodeoxycholic acid (UDCA), cholic acid (CA), glycochenodeoxycholic acid (GCDCA), glycodeoxycholic acid (GDCA), glycocholic acid (GCA), and deoxycholic acid -d4 (d4-DCA) were purchased from Sigma–Aldrich (St. Louis, MO, USA). 5ß-Cholanic acid-3ß, 12a-diol, hyocholic acid (HCA), glycoursodeoxycholic acid (GUDCA), and glycohyocholic acid (GHCA) were purchased from Steraloids (Newport, RI, USA). 5-Cholenic acid-3ß-ol, 5ß-cholenic acid-7a-ol-3-one, and isolithocholic acid (iso-LCA) were purchased from Toronto Research Chemicals (Toronto, ON, Canada); tauroursodeoxycholic acid (TUDCA) was purchased from Cayman Chemical (Ann Arbor, MI, USA). Glycocholic acid-d4 (d4-GCA) and taurochenodeoxycholic acid-d4 (d4-TCDCA) were purchased from Cambridge Isotope Laboratories, Inc. (Tewksbury, MA, USA). LC-/MS-grade methanol, acetonitrile, isopropanol, and formic acid were purchased from Merck Millipore (Burlington, MA, USA).

Calibrators were prepared from a methanolic stock solution containing a final concentration of 5000 ng/mL of each standard and were diluted to fourteen levels (0.025 ng/mL, 0.05 ng/mL, 0.125 ng/mL, 0.25 ng/mL, 0.5 ng/mL, 1.25 ng/mL, 2.5 ng/mL, 5 ng/mL, 12.5 ng/mL, 25 ng/mL, 50 ng/mL, 125 ng/mL, 250 ng/mL, and 500 ng/mL) with 75% methanol, each containing 25 ng/mL of internal standards (d4-DCA, d4-GCA, and d4-TCDCA). Quality control samples were prepared by spiking low, middle, and high levels of standard mixer in a pooled plasma with final levels of 2.5 ng/mL, 12.5 ng/mL, and 50 ng/mL.

For the bile acid analysis, 300 μL of methanol containing internal standards (deoxycholic acid-d4, glycocholic acid-d4, and taurochenodeoxycholic acid) was added to 100 μL of plasma for liquid–liquid extraction. The mixture was kept on ice for 30 min and then centrifuged at 12,000 rpm for 30 min at 4 °C for protein precipitation. The supernatant was transferred to a sample vial and analyzed using an LCMS system (UPLC with Xevo TQS MS, Waters, Manchester, UK) with a negative electrospray ionization mode and multiple reaction monitoring (Supplementary Figure S1). Chromatographic separation was performed on an Acquity BEH C8 reversed-phase column (2.1 × 100-mm i.d., 1.7 μm, Waters Corp., Milford, MA, USA) at 60 °C with mobile phase A (10% acetonitrile with 0.01% formic acid) and mobile phase B (acetonitrile/isopropanol, 1:1, *v*/*v* with 0.01% formic acid) at a flow rate of 0.6 mL/min. The gradient profile was as follows: 10% B for 0.1 min, linear gradient 10–35% B over 9.15 min, 35–85% B over 2.25 min, 85–99% B over 0.5 min, and 99% B for 0.5 min, followed by re-equilibration for 3 min. Compound-specific parameters and source parameters were optimized to obtain a maximal signal response with direct infusion. The MS conditions were set as follows: capillary voltage 1.5 kV; desolvation gas flow 1000 L/h; desolvation temperature 600 °C; source temperature 150 °C; and voltage 60 V. System operation and data acquisition were managed using Mass Lynx software version 4.2.

Quality controls were used to evaluate precision during analysis, and the metabolic data were analyzed by TargetLynx (Waters, Milford, MA, USA) using the area under the curve of each analyte. Linearity was evaluated using the calibration curve with linear regression; the weight concentration was then converted into molar concentration according to the molecular weight of each bile acid.

Originally, 21 BAs were included for analysis. The BAs’ profiles went through quality control checks. We found that 5 metabolites, including 4 taurine-conjugated BAs, did not pass the quality control criteria. Therefore, the included 16 BAs are more glycine-conjugated than taurine-conjugated.

### 2.4. Statistical Analysis

Continuous variables were summarized using mean and standard deviation (SD), or standard error (SE). Group comparisons were conducted using the Mann–Whitney U test or Kruskal–Wallis test with Dunn’s *post hoc* test, incorporated with the Benjamini–Hochberg test to control the false discovery rate when appropriate. Categorical variables, presented as counts and percentages, were analyzed using Fisher’s exact test. A Pearson correlation analysis was used to investigate the associations between metabolite levels and clinical parameters. To assess the diagnostic potential of individual metabolites in distinguishing HD patients from HCs, a receiver operating characteristic (ROC) analysis was conducted. Promising metabolites were selected and further analyzed using a support vector machine (SVM) algorithm, with model performance evaluated through ROC curve generation via Monte-Carlo cross-validation with balanced sub-sampling. Smoothed ROC curves were created based on 100 cross-validation iterations. All statistical analyses were performed using R software version 4.0.3, utilizing the rstatix and metaboanalyst packages (R Foundation, Jaunpur, India).

## 3. Results

**Figure 1:**
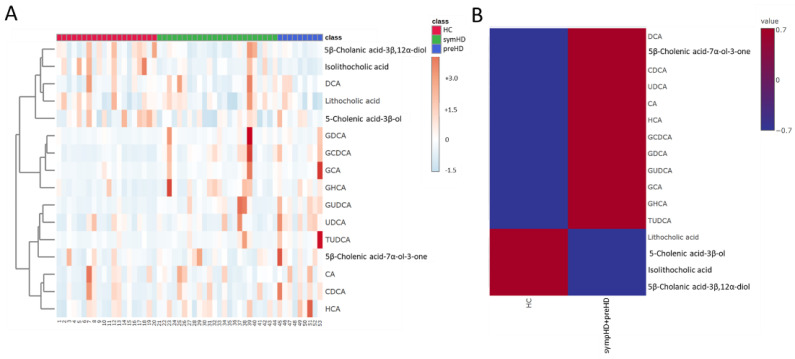
(**A**) Hierarchical clustering heatmap displaying candidate bile acids. The dendrogram on the left side represents the clustering of these bile acids. The colors on top of the heatmap correspond to the following different groups: healthy controls (HC), patients with presymptomatic Huntington’s disease (preHD), and symptomatic Huntington’s disease (symHD). The colors in the heatmap indicate normalized intensities, scaled to a mean of zero and unit variance for each feature. (**B**) Group averages of candidate bile acids, with merged data from HD and preHD. Abbreviations: HCA, hyocholic acid; CDCA, chenodeoxycholic acid; CA, cholic acid; GUDCA, glycoursodeoxycholic acid; TUDCA, tauroursodeoxycholic acid; UDCA, ursodeoxycholic acid; GHCA, glycohyocholic acid; GCA, glycocholic acid; GCDCA, glycochenodeoxycholic acid; GDCA, glycodeoxycholic acid; DCA, deoxycholic acid.

This study recruited a cohort of 33 genetically confirmed HD patients (24 with symHD and 9 preHD) and 20 HCs (Table 1). We quantified the plasma concentrations of 16 BAs using LCMS (Table 2). In HD patients, levels of isolithocholic acid (iso-LCA) were significantly lower (HD vs. HC: 7.81 ± 1.50 nM vs. 18.78 ± 3.57 nM, *p* = 0.022), while levels of glycochenodeoxycholic acid (GCDCA) (HD vs. HC: 2074.67 ± 369.74 nM vs. 580.02 ± 95.33 nM, *p* < 0.001) and glycoursodeoxycholic acid (GUDCA) (HD vs. HC: 313.45 ± 68.64 nM vs. 74.39 ± 16.56 nM, *p* = 0.009) were elevated compared to HC. The hierarchical clustering heatmap of the selected metabolites is displayed in Figure 1, showing most symHD and preHD patients clustered together. However, the levels of these metabolites did not correlate with UPDRS scores or disease burden.

**Figure 2:**
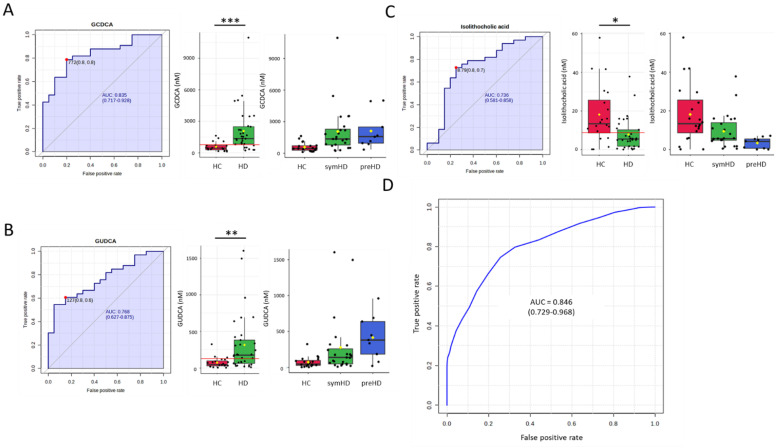
Receiver operating characteristic (ROC) curves and box plots comparing plasma levels of (**A**) glycochenodeoxycholic acid (GCDCA), (**B**) glycoursodeoxycholic acid (GUDCA), and (**C**) isolithocholic acid between patients with Huntington’s disease (HD), symptomatic Huntington’s disease (symHD), presymptomatic Huntington’s disease (preHD), and healthy controls (HC). The shaded area under the ROC curve (AUC) represents the performance in distinguishing HD from HC. The red dots and lines indicate the optimal cut-off points that maximize the sensitivity and specificity of the metabolites for discriminating HD from HC. In the box plots, the black dots represent the metabolite level of each sample, the black center line represents the median, while the red, green, or blue boxes indicate the 25th to 75th percentiles. The black whiskers mark the 5th and 95th percentiles, and the mean values are represented by yellow diamonds. * *p* < 0.05, ** *p* < 0.01, *** *p* < 0.001, statistically significant between two groups (Mann–Whitney U test with Benjamini–Hochberg test). (**D**) ROC analysis of the three bile acids using a support vector machine. One hundred cross-validations were conducted, and the results were averaged to generate the plot. Abbreviations: GCDCA, glycochenodeoxycholic acid; GUDCA, glycoursodeoxycholic acid.

To assess the potential diagnostic utility of the identified BAs as biomarkers for HD, an ROC curve analysis was conducted. GCDCA had the highest area under the ROC curve (AUC) for distinguishing the patients with HD from the HC (AUC = 0.835), followed by GUDCA (AUC = 0.768) and iso-LCA (AUC = 0.736). Employing an SVM algorithm that incorporated GCDCA, GUDCA, and iso-LCA yielded an AUC of 0.84 in distinguishing HD patients from HC (Figure 2 and Table 3). Furthermore, GCDCA showed the highest AUC for distinguishing symHD patients from HC (AUC = 0.828), followed by GUDCA (AUC = 0.738) and iso-LCA (AUC = 0.696). The SVM algorithm using these three biomarkers achieved an AUC of 0.809 in distinguishing symHD patients from HC (Supplementary Figure S2 and Table 3). GUDCA and iso-LCA exhibited the highest AUC for distinguishing preHD patients from HC (AUC = 0.878), followed by GCDCA (AUC = 0.872). These three biomarkers with the SVM algorithm yielded an AUC of 0.888 in differentiating preHD patients from HCs (Supplementary Figure S3 and Table 3). Iso-LCA (AUC = 0.755) and GUDCA (AUC = 0.713) demonstrated reliable performance in distinguishing symHD from preHD (Supplementary Figure S4 and Table 3). These findings suggest the potential of developing a machine-learning algorithm utilizing BA profiles for HD diagnosis.

**Figure 3:**
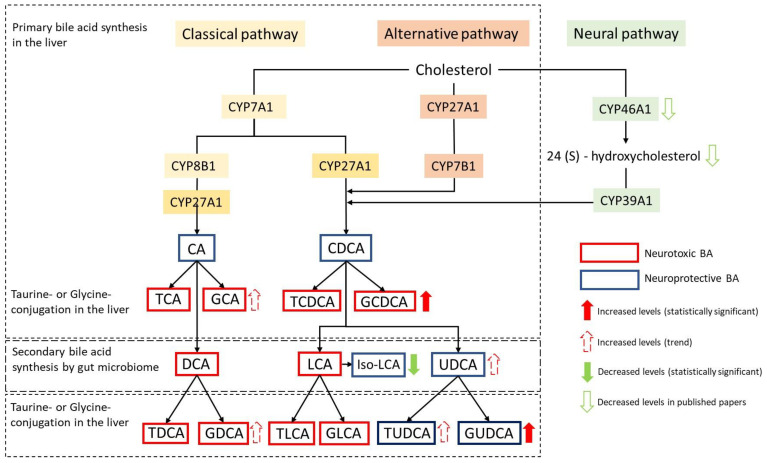
Schematic representation of the alterations of bile acids involved in the synthesis pathway in HD. Primary bile acids are converted to secondary bile acids by the gut microbiome. 24(S)-hydroxycholesterol from the brain mainly involves the alternative pathway. GCDCA and GUDCA levels are elevated and iso-LCA decreased in Huntington’s disease patients (HD) (presymptomatic HD (preHD) + symptomatic HD (symHD), and the total of neurotoxic bile acids (GCA + GDCA + GCDCA) is higher in symHD compared to healthy controls (HC). Conversely, the sum of neuroprotective BAs (UDCA + GUDCA + TUDCA) is increased in preHD carriers compared to HC. Also, it is noted that decreased CYP46A1 and 24(S)-hydroxycholesterol levels in HD have been shown in previously published papers. Abbreviations: CA, cholic acid; CDCA, chenodeoxycholic acid; TCA, taurocholic acid; GCA, glycocholic acid; TCDCA, taurochenodeoxycholic acid; GCDCA, glycochenodeoxycholic acid; DCA, deoxycholic acid; LCA, lithocholic acid; UDCA, ursodeoxycholic acid; TDCA, taurodeoxycholic acid; GDCA, glycodeoxycholic acid; TUDCA, tauroursodeoxycholic acid; GUDCA, glycoursodeoxycholic acid; Iso-LCA, Isolithocholic acid.

To elucidate the specific enzymatic mechanisms underlying the observed alterations in BA metabolism associated with HD, we examined several distinctive ratios indicative of enzymatic processes within both hepatic and gut microbiome environments (Figure 3). We also compared the sum and ratio of several known neuroprotective and neurotoxic BAs to evaluate the dynamic profile of BAs in HD progression (Table 4), as follows:

1. CDCA/CA Ratio: This ratio was used to assess potential shifts in BA synthesis, indicating deviations from the classical to the alternative BA pathway;
2. Ratios of secondary to primary BAs (DCA/CA, GDCA/CA): By assessing these ratios, we investigated disparities in enzymatic activities of the gut microbiome that may lead to increased secondary BA production;
3. Sum of neuroprotective BAs [UDCA + GUDCA + tauroursodeoxycholic acid (TUDCA)]: This sum was used to evaluate the overall neuroprotective effect of BAs;
4. Sum of neurotoxic BAs [glycocholic acid (GCA) + GDCA + GCDCA]: This sum was used to assess the magnitude of neurotoxicity of BAs;
5. Ratio of UDCA + GUDCA + TUDCA/GCA + GDCA + GCDCA: This ratio indicates if neuroprotective BAs are proportionally higher than neurotoxic BAs in HD.

The ratio of alternative to classical BAs (CDCA/CA) was highest in preHD (4.17 ± 1.23) compared to HC (3.08 ± 0.51) and symHD (3.18 ± 0.44). The ratio of GDCA/CA (i.e., the conversion of primary to secondary BAs) in symHD (13.45 ± 4.12) showed an upward trend compared to HC (4.86 ± 1.44, *p* = 0.06), although it was not statistically significant. The levels of neuroprotective BAs (UDCA + GUDCA + TUDCA) were significantly elevated in preHD (698.07 ± 161.76 nM) compared to HC (186.75 ± 34.42, *p* = 0.013). Conversely, the levels of neurotoxic BAs (GCA + GDCA + GCDCA) were significantly elevated in symHD (3471.79 ± 1017.3 nM) compared to HC (970.16 ± 179.78 nM, *p* = 0.023) (Table 4). Although some of the ratios were not statistically significant, the results provide insight into the complex enzymatic processes contributing to BA profile alterations in HD.

## 4. Discussion

We report significant alterations in four of the 16 primary and secondary BAs examined in the plasma of preHD and symHD patients. Specifically, iso-LCA levels were reduced in HD patients, while GCDCA and GUDCA were elevated compared to HC. These changes suggest a disturbance in the brain–gut axis, similar to what is seen in other neurodegenerative disorders such as AD, PD, and ALS [20,21]. The aggregation of mutant HTT protein, present not only in the brain but also in the gastrointestinal tract and liver cells, may lead to the disruption of normal hepatic function and the gut microbiome [32,33]. Indeed, gut dysbiosis can occur in patients with HD, and some genera of microbiota have been shown to correlate with specific clinical features; lactobacillus is negatively correlated with the mini-mental state examination score [34]. Gut dysbiosis has also been reported in the R6/2 HD mouse model [35]. A study has shown that mutant huntingtin interferes with sterol regulatory element-binding transcription factor 2 (SREBP2) nuclear translocation to prevent the SREBP2-mediated transcription of genes involved in the synthesis and turnover of cholesterol in the brain [36], which leads to decreased 24 (S)hydroxycholesterol (a precursor of CDCA) levels in the brain and peripheral tissue and blood [37]. The knockout of huntingtin in hepatocytes is associated with altered cholesterol and total bile acid levels and the decreased expression of genes involved in bile acid synthesis [38]. Since secondary BA production and BA deconjugation in humans rely entirely on gut microbes, disruptions in the microbiome (gut dysbiosis) and disturbed cholesterol synthesis and turnover could influence the BA profile alterations seen in HD.

Studies have reported that both unconjugated and conjugated BAs can cross the blood–brain barrier (BBB) [39,40,41,42]. Unconjugated BAs readily diffuse across the BBB, with brain levels reflecting serum levels [43], while conjugated BAs require active transport due to their structural and hydrophilicity [44]. The hydrophobicity indices of BAs influence their ability to cross the BBB, with more hydrophilic BAs being more neuroprotective (Table 5) [45,46]. Among the hydrophilic BAs, CA and CDCA are neuroprotective, while UDCA, its glycine-conjugated form GUDCA, and its taurine-conjugated form TUDCA are considered the most hydrophilic and neuroprotective. These neuroprotective BAs, particularly UDCA and TUDCA, have shown potential as therapeutic agents for neurodegenerative disorders, such as in AD and PD animal models, due to their anti-neuroinflammatory, oxidative stress-reducing, apoptotic cascade regulating, mitochondrial protecting, and protein stabilizing effects [47,48,49,50,51]. For instance, in a study with AD rat models, daily CDCA injections significantly improved cognitive and spatial performance to levels similar to control subjects [52]. Conversely, GCA, GDCA, and GCDCA are regarded as the most hydrophobic and neurotoxic BAs. These studies highlight the important role BAs may play in the pathogenesis and treatment of neurodegenerative diseases.

To the best of our knowledge, direct studies of BA profiles in patients with HD have not yet been reported, although altered total BAs have been shown in a mouse model of the knockout of huntingtin [38], and TUDCA has been applied to HD mice to show neuroprotection effects [53,54,55]. Our study is the first to show altered BA metabolite profiles in the plasma of HD patients. Similar to findings in AD [22], the ratios of GDCA/CA in our HD patients tended to increase compared to HCs, suggesting gut dysfunction that may cause a shift from primary BAs to secondary BAs. Furthermore, higher GCDCA levels were associated with HD, and the sum of neurotoxic BAs (GCA + GDCA + GCDCA) was associated with symHD. Conversely, cytoprotective GUDCA was elevated in the HD group, particularly in preHD, indicating a compensatory response to cytotoxicity. These results suggest that altered BA metabolism may contribute to HD pathogenesis (Figure 3).

While studies specifically addressing the roles of BAs in HD are limited, some have demonstrated the neuroprotective effects of TUDCA in 3-nitropropionic acid or transgenic HD models [53,54,55]. One study found a correlation between plasma levels of 24S-hydroxycholesterol (24OHC) and a significant caudate volume reduction during the transition from pre-HD to symHD [28]. Since nearly all circulating 24OHC originates in the brain [56,57,58], this has led to the hypothesis that peripheral levels of 24OHC may reflect CNS cholesterol turnover [59,60]. During metabolism, 24OHC is converted to BAs via the alternative pathway, predominantly resulting in CDCA (Figure 3) [61]. This suggests that CDCA, derived from 24OHC, might activate neuroprotective pathways in response to neuronal damage, altering BA metabolite levels in the brain and plasma. Notably, our study found elevated levels of CDCA (Table 2) and the ratio of CDCA/CA in preHD (Table 4), indicating a potential shift in BA synthesis from the classical to the alternative pathway, possibly driven by increased brain cholesterol metabolism during neurodegeneration [59]. An unpublished study also found mildly increased 24OHC levels in young preHD individuals [60], possibly indicating a compensatory response to neuronal cell membrane damage. This compensatory response may also activate pathways that enhance neuroprotective BAs (UDCA + GUDCA + TUDCA) (Table 4) and reduce primary to secondary BA conversion (DCA/CA) to mitigate neurodegenerative processes in preHD. However, as the disease progresses to symHD, this compensatory response diminishes, and reduced plasma 24OHC was found to correlate with the degree of caudate atrophy [28]. Although some of the observed ratios did not reach statistical significance, potentially due to a small sample size, these findings suggest that altered BA enzymatic activities during preHD may have potential diagnostic value. These observations, although not specific to HD, address the potential interconnection between cholesterol turnover, BAs, and neuroprotective mechanisms in the context of HD.

Our study also indicates that GCDCA, GUDCA, iso-LCA, or their combination can differentiate HD, symHD, or preHD patients from HC. Furthermore, GUDCA or iso-LCA can distinguish symHD from preHD carriers, suggesting their potential as biomarkers for disease status. A previous study showed that iso-LCA contributes to protection against enteropathogenic infection and is significantly increased in centenarians [62], suggesting its role in gut microbiota-mediated neurodegeneration. However, the functional roles of iso-LCA remain to be explored. Future research should aim to elucidate the complex interactions between BAs and the brain–gut axis, contributing to our understanding of their roles in the pathogenesis of neurodegeneration in HD.

### Limitations and Future Directions

This study has several limitations that warrant consideration. Foremost among these is the small sample size, which may compromise the statistical power of our findings. As an observational study, potential confounding variables, such as medication usage, dietary habits, age, and gender, may have influenced the observed disparities in BA profiles between groups. In addition, five BAs have been removed from the analysis due to not passing quality control criteria, which may miss some of the metabolites that may demonstrate significant differences between HD and HC. To enhance the robustness of our findings and fully elucidate the role of BAs in HD, future investigations should prioritize larger sample sizes and include independent validation sets of HD patients. Longitudinal studies are imperative to uncover temporal patterns and establish causal relationships. This approach will contribute to a more precise understanding of the clinical implications of alterations in BA profiles in HD, thus advancing our knowledge in this area of research.

## 5. Conclusions

Emerging insights into the alterations of BA profiles across various neurodegenerative disorders underscore the potential regulatory roles exerted by BAs within the brain. Our comprehensive analysis has revealed significant perturbations in three specific BAs in both preHD and symHD patients compared to HC, which have not been reported before. These novel findings highlight the potential involvement of BAs in the pathogenesis of HD and exemplify the need for future investigations focusing on BAs. Such endeavors may identify novel biomarkers for diagnostic purposes and unveil promising pharmacological targets to ameliorate the progression of HD.

## Figures and Tables

**Table 1:** Demographic characteristics and blood biochemical parameters of the patients with Huntington’s disease (HD), presymptomatic (preHD) carriers, symptomatic Huntington’s disease (symHD), and healthy controls (HC).

	HC	HD		
	(*n* = 20)	preHD (*n* = 9)	symHD (*n* = 24)	All (*n* = 33)
Age (years)	52.7 ± 9.0	32.0 ± 7.9 *	50.0 ± 10.9	45.1 ± 13.1
Male (%)	10 (50.00)	1 (11.1) **	16 (66.7)	17 (51.5)
BMI	23.4 ± 2.6	20.2 ± 2.4	21.9 ± 2.5	21.4 ± 2.6
Pre-prandial glucose (mg/dL)	93.3 ± 10.7	87.8 ± 7.9	98.3 ± 16.6	96.7 ± 17.2
UHDRS				
Total motor score		0	39.2 ± 19.3	
Independence scale		100	66.7 ± 25.9	
Functional capacity		13	6.4 ± 4.2	
Disease burden		272.6 ± 182.3	457.5 ± 124.6	
Medications				
Tetrabenazine (%)	0	0	6 (25.00)	
Antipsychotics (%)	0	0	11 (45.9)	
Antidepressants (%)	0	0	8 (33.3)	
Benzodiazepines (%)	3 (15.00)	0	14 (58.3)	
Ubidecarenone (%)	0	0	10 (41.7)	

BMI, body mass index; HC, healthy controls; HD, Huntington’s disease; preHD, presymptomatic Huntington’s disease; symHD, symptomatic Huntington’s disease; UHDRS, Unified Huntington’s Disease Rating Scale. Data are expressed as mean and standard deviation. * Statistically significant in comparison with HC or symHD, *p* < 0.05, Kruskal–Wallis test with Dunn’s *post hoc* test. ** Statistically significant in comparison with HC or symHD, *p* < 0.05, Fisher’s exact test.

**Table 2:** Levels of 16 different bile acids in plasma of the patients with Huntington’s disease (HD), presymptomatic carriers (preHD), symptomatic Huntington’s disease (symHD), and healthy controls (HC).

Metabolite Name (nM)	HC (*n* = 20)	HD		
		preHD (*n* = 9)	symHD (*n* = 24)	All (*n* = 33)
5-Cholenic acid-3ß-ol	6.90 ± 2.82	6.54 ± 2.11	5.27 ± 1.91	5.62 ± 2.01
Lithocholic acid (LCA)	17.02 ± 9.03	12.88 ± 8.84	17.05 ± 9.53	15.90 ± 9.38
Isolithocholic acid (Iso-LCA)	18.78 ±15.55	3.37 ± 2.91	9.72 ± 9.57	7.81 ± 8.62 *
5ß-Cholenic acid-7a-ol-3-one	1.21 ± 0.1.23	2.38 ± 2.53	1.34 ± 1.24	1.62 ± 1.71
5ß-Cholanic acid-3ß, 12a-diol	53.39 ± 42.77	31.58 ± 14.88	38.68 ±38.94	37.21 ± 35.2
Deoxycholic acid (DCA)	341.78 ± 340.09	388.33 ± 308.75	429.91 ± 419.96	421.31 ± 394.83
Chenodeoxycholic acid (CDCA)	326.70 ± 423.08	652.67 ± 575.75	346.15 ± 318.6	429.75 ± 418.38
Ursodeoxycholic acid (UDCA)	107.38 ± 104.4	247.76 ± 176.28	120.37 ± 130.07	155.11 ± 152.47
Cholic acid (CA)	155.51 ± 218.78	219.84 ± 211.44	153.75 ± 164.29	171.78 ± 177.4
Hyocholic acid (HCA)	9.95 ± 7.77	19.24 ± 17.67	12.31 ± 8.15	14.20 ± 11.64
Glycochenodeoxycholic acid (GCDCA)	580.02 ± 426.35	2110.80 ± 1731.74	2061.12 ± 2287.53	2074.67 ± 2123.98 *
Glycodeoxycholic acid (GDCA)	195.36 ± 220.9	591.26 ± 962.54	893.26 ± 1880.57	810.89 ± 1670.98
Glycoursodeoxycholic acid (GUDCA)	74.39 ± 74.08	414.84 ± 306.45	275.43 ± 422.03	313.45 ± 394.29 *
Glycocholic acid (GCA)	194.78 ± 268.59	903.47 ± 1777.84	517.41 ± 931.16	622.70 ± 1201.61
Glycohyocholic acid (GHCA)	11.15 ±12.37	21.86 ± 13.34	25.98 ± 29.53	24.86 ± 25.97
Tauroursodeoxycholic acid (TUDCA)	4.98 ± 6.73	35.47 ± 54.85	11.26 ± 20.97	17.86 ± 34.47

Data are expressed as mean and standard deviation. * *p* < 0.05, statistically significant in comparison with HC, Mann–Whitney U test with Benjamini–Hochberg test.

**Table 3:** The receiver operating characteristic (ROC) curve analysis; summary of the area under the curve (AUC) values of three bile acids (BAs) that were significant (higher than 0.8) in distinguishing between the patients with Huntington’s disease (HD), presymptomatic (preHD) carriers, symptomatic Huntington’s disease (symHD), and healthy controls (HC).

	GCDCA	GUDCA	iso-LCA	SVM Algorithm
HD vs. HC	0.835 *	0.768	0.736	0.846
symHD vs. HC	0.828 *	0.738	0.696	0.809
preHD vs. HC	0.872	0.878 *	0.878 *	0.888
preHD vs. symHD	-	0.713	0.755 *	-

* Highest AUC among the three BAs. Abbreviations: GCDCA, glycochenodeoxycholic acid; GUDCA, glycoursodeoxycholic acid; iso-LCA, isolithocholic acid; SVM, support vector machine.

**Table 4:** Ratios of bile acids (BA) reflective of the gut microbiome and liver enzymatic activities, and the total amount of neuroprotective and neurotoxic BA in the patients with Huntington’s disease (HD), presymptomatic carriers (preHD), symptomatic Huntington’s disease (symHD), and healthy controls (HC).

Informative about Metabolic Process	Ratios or Total Amounts (nM)	HC	preHD	symHD
Bile acid synthesis: alternative vs. classical pathway	CDCA/CA	3.08 ± 2.27	4.17 ± 3.70	3.18 ± 2.15
Conversion from primary to secondary BA by the gut microbiome	DCA/CA GDCA/CA	7.53 ± 8.44 4.86 ± 6.44	4.06 ± 4.04 5.35 ± 5.96	5.82 ± 6.11 13.45 ± 20.19
Neuroprotective BA	UDCA + GUDCA + TUDCA	186.75 ± 153.95	698.07 ± 485.28 *	407.05 ± 532.92
Neurotoxic BA	GCA + GDCA + GCDCA	970.16 ± 804.02	3605.53 ± 4034.86	3471.79 ± 4983.96 *
Neuroprotective BA/ Neurotoxic BA	UDCA + GUDCA + TUDCA/ GCA + GDCA + GCDCA	0.31 ± 0.38	0.25 ± 0.17	0.19 ± 0.17

Data are expressed as mean and standard deviation; * *p* < 0.05, statistically significant in comparison with HC, Kruskal–Wallis test with Dunn’s *post hoc* test. Abbreviations: CDCA, chenodeoxycholic acid; CA, cholic acid; DCA, deoxycholic acid; GDCA, glycodeoxycholic acid; GCDCA, glycochenodeoxycholic acid; UDCA, ursodeoxycholic acid; GUDCA glycoursodeoxycholic acid; TUDCA, tauroursodeoxycholic acid.

**Table 5:** Summary of bile acids based on their hydrophilicity.

Considered to be neurotoxic BA	GCA, GDCA, GCDCA	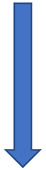 Hydrophilicity
	GHCA, HCA	
	LCA	
	DCA	
Considered to be neuroprotective BA	CDCA	
	CA	
	UDCA, GUDCA, TUDCA	

The more hydrophobic bile acids are represented by their acronyms at the top of the table, and the more hydrophilic bile acids are at the bottom part of the table. Abbreviations: GCA, glycocholic acid; GDCA, glycodeoxycholic acid; GCDCA, glycochenodeoxycholic acid; GHCA, glycohyocholic acid; HCA, hyocholic acid; LCA, lithocholic acid, DCA, deoxycholic acid; CDCA, chenodeoxycholic acid; CA, cholic acid; UDCA, ursodeoxycholic acid; GUDCA, glycoursodeoxycholic acid; TUDCA, tauroursodeoxycholic acid.

## Data Availability

The data presented in this study are available on request from the corresponding author due to privacy.

## Supplementary Materials

The following supporting information can be downloaded at https://www.mdpi.com/article/10.3390/metabo14070394/s1: Figure S1: The chromatograms of 16 bile acids in the plasma samples and standards; Figure S2: Receiver operating characteristic (ROC) curves of (A) glycochenodeoxycholic acid (GCDCA), (B) glycoursodeoxycholic acid (GUDCA), and (C) isolithocholic acid between patients with symptomatic Huntington’s disease (symHD) and healthy controls (HC); Figure S3: Receiver operating characteristic (ROC) curves of (A) glycochenodeoxycholic acid (GCDCA), (B) glycoursodeoxycholic acid (GUDCA), and (C) isolithocholic acid between patients with presymptomatic Huntington’s disease (preHD) and healthy controls (HC); Figure S4: Receiver operating characteristic (ROC) curves of (A) isolithocholic acid and (B) glycoursodeoxycholic acid (GUDCA) between patients with presymptomatic Huntington’s disease (preHD) and symptomatic Huntington’s disease (symHD).
