# Supplementary material for: Diagnostic Potential of Alternations of Bile Acid Profiles in the Plasma of Patients with Huntington’s Disease
Source: Metabolites. 2024 Jul 20;14(7):394. doi: 10.3390/metabo14070394 (PMC11278952; doi:10.3390/metabo14070394)
Supplement: Supplementary file 1 [file metabolites-14-00394-s001.zip › metabolites-3076246-supplementary.pdf]

Supplementary Materials

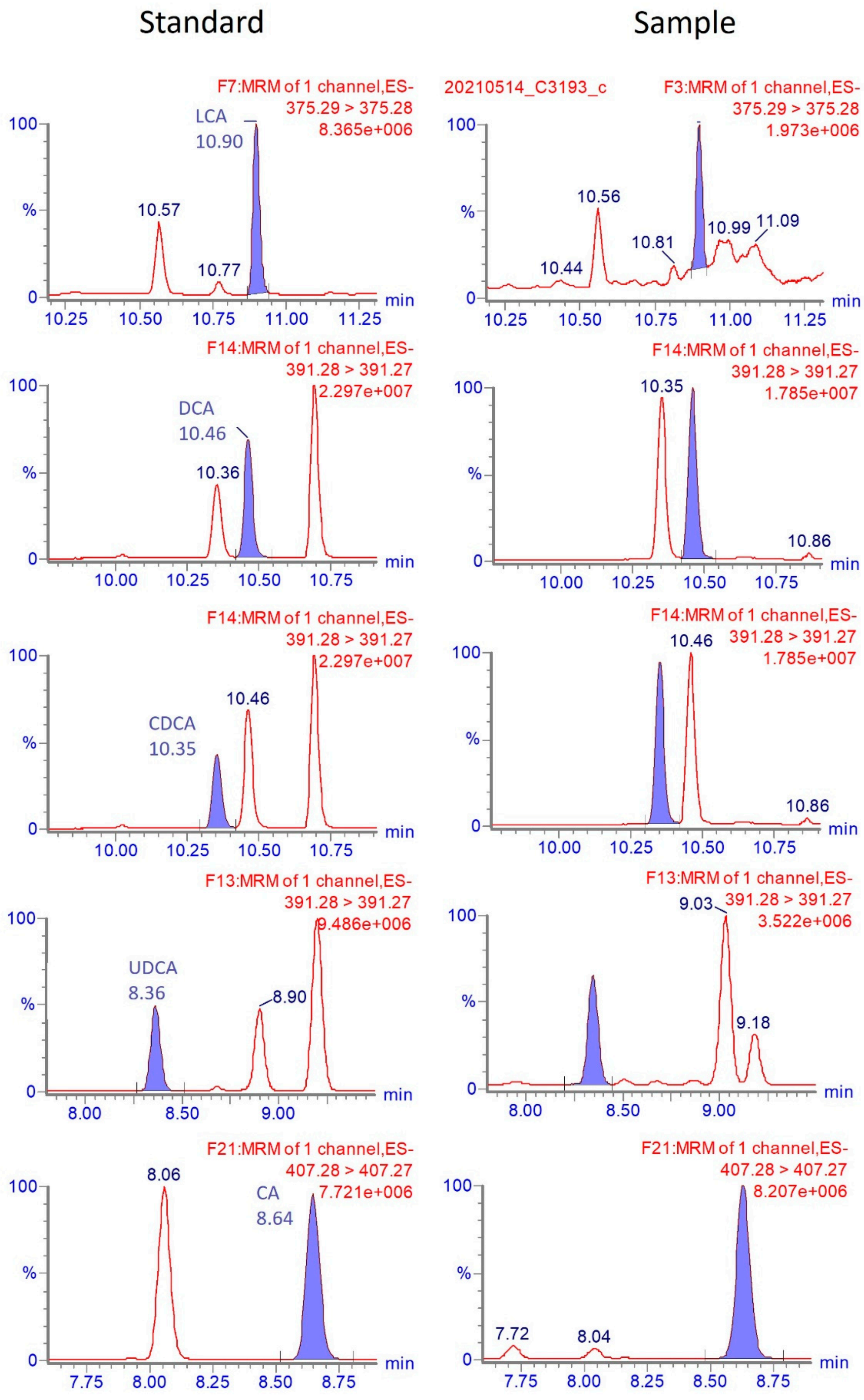

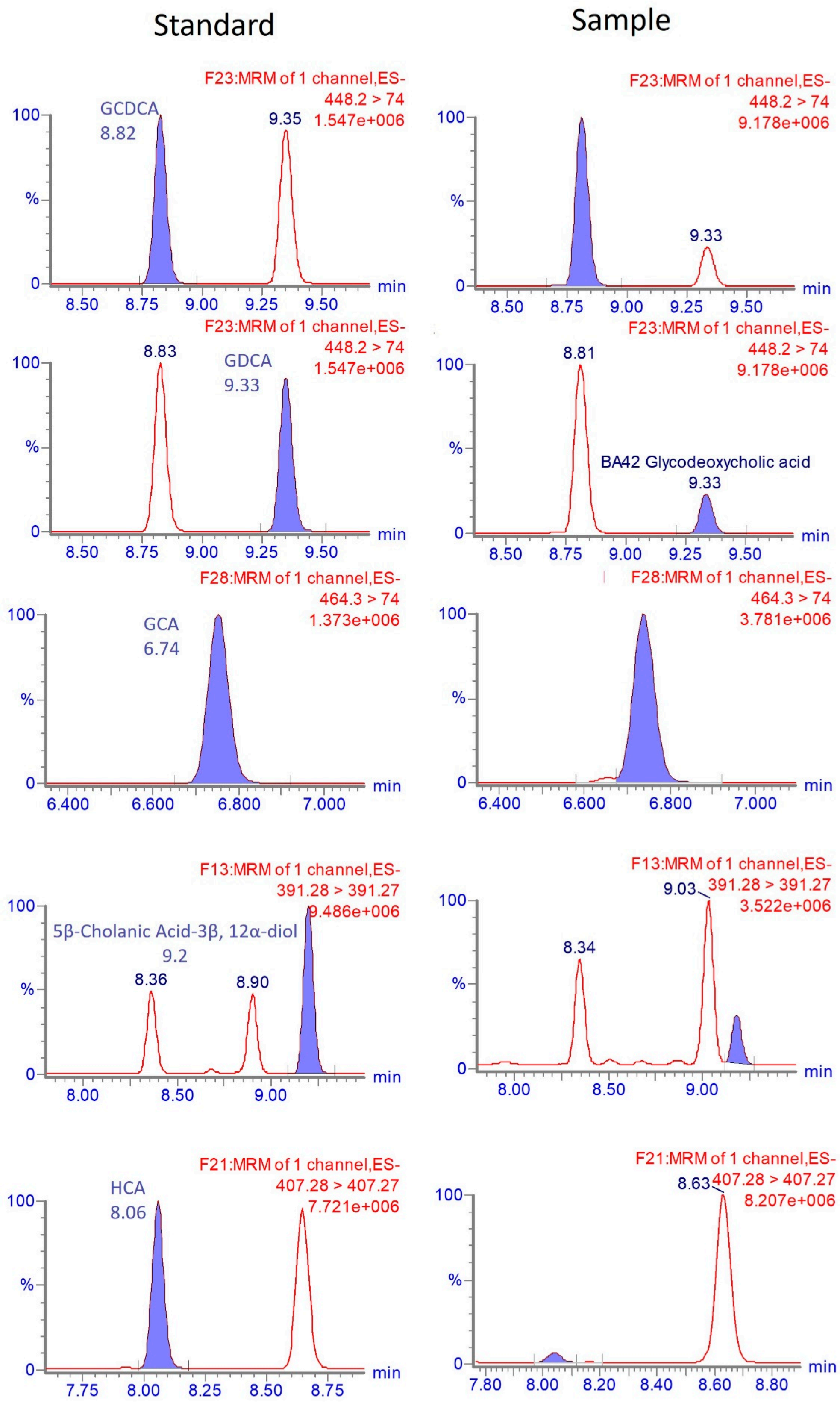
Standard
Sample
F23:MRM of 1 channel,ES-
448.2 > 74
1.547e+006
GCDCA
8.82
9.35
F23:MRM of 1 channel,ES-
448.2 > 74
9.178e+006
9.33
F23:MRM of 1 channel,ES-
448.2 > 74
1.547e+006
8.83
GDCA
9.33
F23:MRM of 1 channel,ES-
448.2 > 74
9.178e+006
8.81
BA42 Glycodeoxycholic acid
9.33
F28:MRM of 1 channel,ES-
464.3 > 74
1.373e+006
GCA
6.74
F28:MRM of 1 channel,ES-
464.3 > 74
3.781e+006
F13:MRM of 1 channel,ES-
391.28 > 391.27
9.486e+006
5β-Cholanic Acid-3β, 12α-diol
9.2
8.36
8.90
F13:MRM of 1 channel,ES-
391.28 > 391.27
3.522e+006
9.03
8.34
F21:MRM of 1 channel,ES-
407.28 > 407.27
7.721e+006
HCA
8.06
F21:MRM of 1 channel,ES-
407.28 > 407.27
8.207e+006
8.63
100
%
0
min

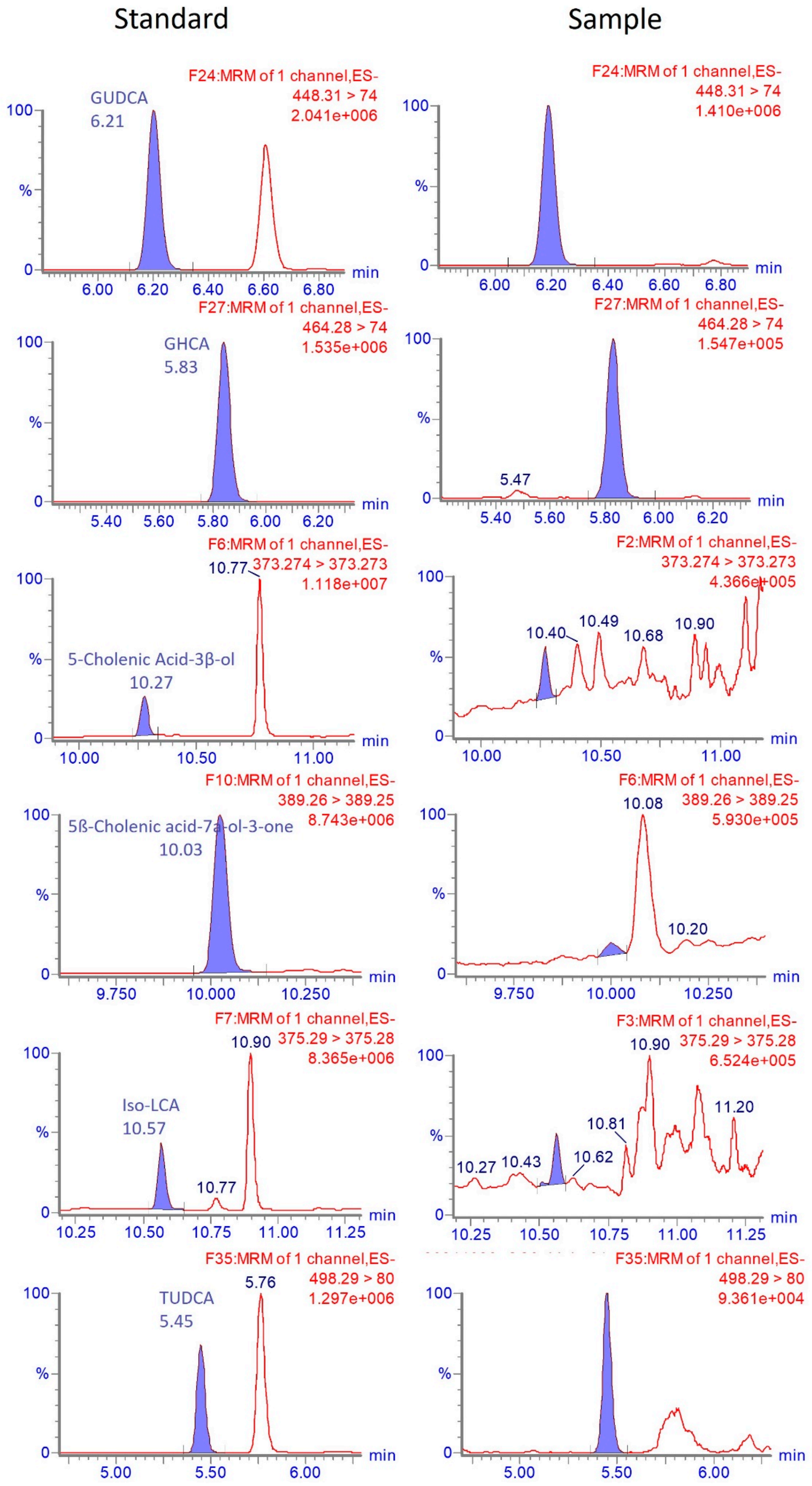

Figure S1. The chromatograms of 16 bile acids in the plasma samples and standards.

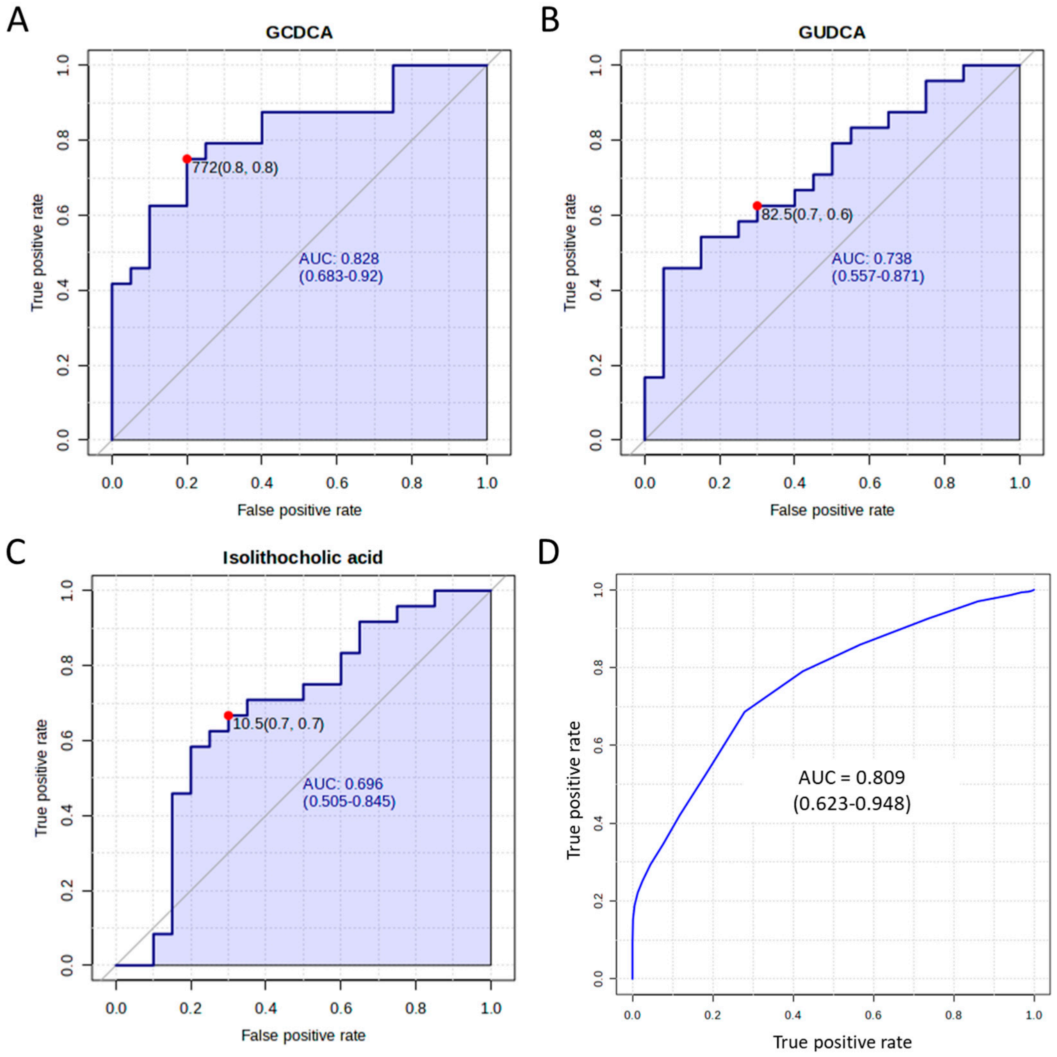

Figure S2. Receiver operating characteristic (ROC) curves of (A) glycochenodeoxycholic acid (GCDCA), (B) glycoursodeoxycholic acid (GUDCA), and (C) isolithocholic acid between patients with symptomatic Huntington's disease (symHD) and healthy controls (HC). The shaded area under the ROC curve (AUC) represents the performance in distinguishing symHD from HC. The red dots indicate the optimal cut-off points that maximize the sensitivity and specificity of the metabolites for discriminating HD from HC. (D) ROC analysis of the three bile acids using a support vector machine. One hundred cross-validations were performed, and the results were averaged to generate the plot. Abbreviations: GCDCA, glycochenodeoxycholic acid; GUDCA, glycoursodeoxycholic acid.

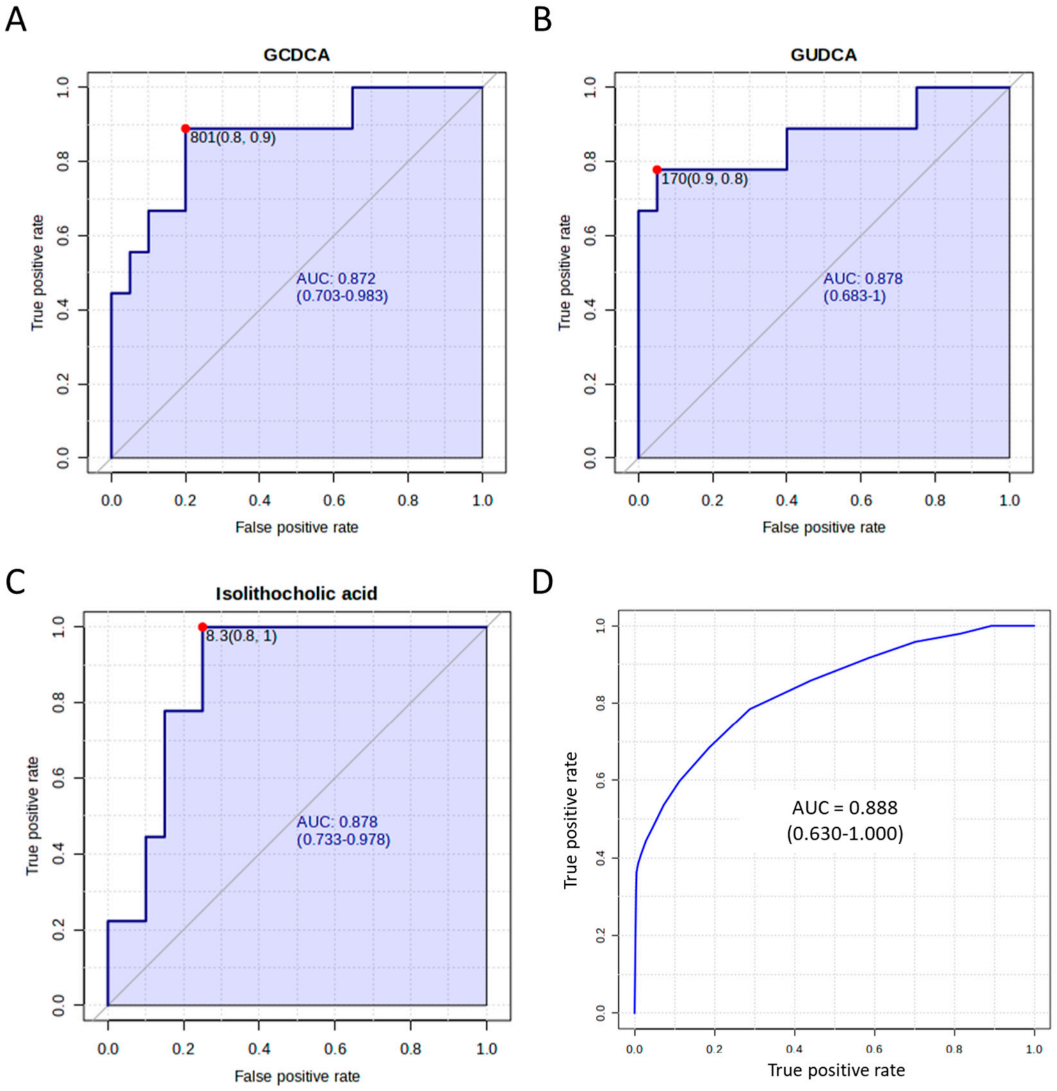

Figure S3. Receiver operating characteristic (ROC) curves of (A) glycochenodeoxycholic acid (GCDCA), (B) glycoursodeoxycholic acid (GUDCA), and (C) isolithocholic acid between patients with presymptomatic Huntington's disease (preHD) and healthy controls (HC). The shaded area under the ROC curve (AUC) represents the performance in distinguishing preHD from HC. The red dots indicate the optimal cut-off points that maximize the sensitivity and specificity of the metabolites for discriminating HD from HC. (D) ROC analysis of the three bile acids using a support vector machine. One hundred cross-validations were performed, and the results were averaged to generate the plot. Abbreviations: GCDCA, glycochenodeoxycholic acid; GUDCA, glycoursodeoxycholic acid.

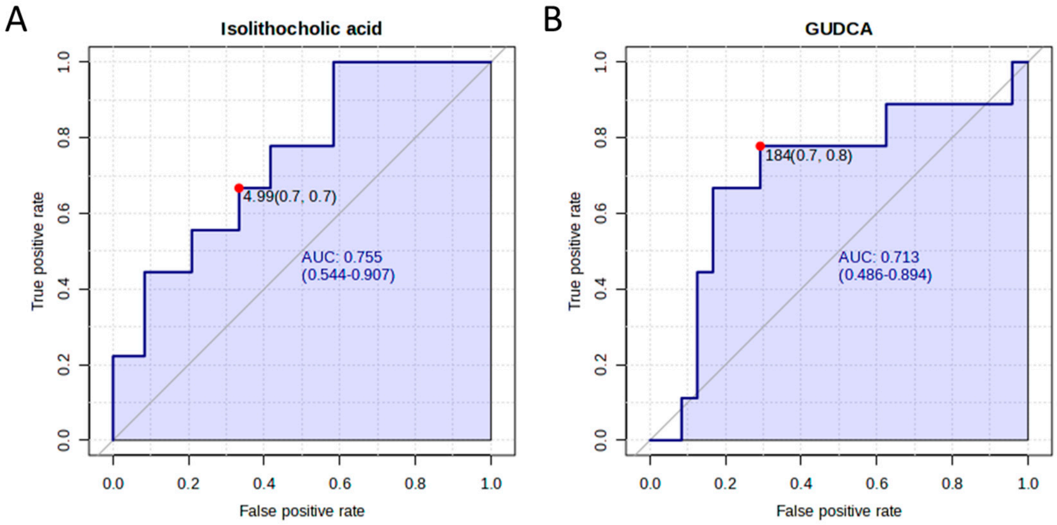

Figure S4. Receiver operating characteristic (ROC) curves of (A) isolithocholic acid and (B) glycoursodeoxycholic acid (GUDCA) between patients with presymptomatic Huntington's disease (preHD) and symptomatic Huntington’s disease (symHD). The shaded area under the ROC curve (AUC) represents the performance in distinguishing preHD from symHD. The red dots indicate the optimal cut-off points that maximize the sensitivity and specificity of the metabolites for discriminating HD from HC. Abbreviations: GUDCA, glycoursodeoxycholic acid.
